# Artificial Intelligence and Suicide Prevention: A Systematic Review of Machine Learning Investigations

**DOI:** 10.3390/ijerph17165929

**Published:** 2020-08-15

**Authors:** Rebecca A. Bernert, Amanda M. Hilberg, Ruth Melia, Jane Paik Kim, Nigam H. Shah, Freddy Abnousi

**Affiliations:** 1 Stanford Suicide Prevention Research Laboratory, Department of Psychiatry and Behavioral Sciences, Stanford University School of Medicine, Stanford, CA 94304, USA; 2 Department of Psychology, National University of Ireland, Galway, Ireland; 3 Department of Medicine, Center for Biomedical Informatics Research, Stanford University School of Medicine, Stanford, CA 94304, USA; 4 Informatics, Stanford Center for Clinical and Translational Research, and Education (Spectrum), Stanford University, Stanford CA 94304, USA; 5 Facebook, Menlo Park, CA 94025, USA; 6 Yale University School of Medicine, New Haven, CT 06510, USA

**Keywords:** artificial intelligence, machine learning, suicide, prediction, risk, intervention

## Abstract

Suicide is a leading cause of death that defies prediction and challenges prevention efforts worldwide. Artificial intelligence (AI) and machine learning (ML) have emerged as a means of investigating large datasets to enhance risk detection. A systematic review of ML investigations evaluating suicidal behaviors was conducted using PubMed/MEDLINE, PsychInfo, Web-of-Science, and EMBASE, employing search strings and MeSH terms relevant to suicide and AI. Databases were supplemented by hand-search techniques and Google Scholar. Inclusion criteria: (1) journal article, available in English, (2) original investigation, (3) employment of AI/ML, (4) evaluation of a suicide risk outcome. N = 594 records were identified based on abstract search, and 25 hand-searched reports. N = 461 reports remained after duplicates were removed, n = 316 were excluded after abstract screening. Of n = 149 full-text articles assessed for eligibility, n = 87 were included for quantitative synthesis, grouped according to suicide behavior outcome. Reports varied widely in methodology and outcomes. Results suggest high levels of risk classification accuracy (>90%) and Area Under the Curve (AUC) in the prediction of suicidal behaviors. We report key findings and central limitations in the use of AI/ML frameworks to guide additional research, which hold the potential to impact suicide on broad scale.

## 1. Introduction

Suicide is a complex, but preventable public health problem that challenges prediction due to its transdiagnostic, yet rare occurrence at the population-level. Beyond the inestimable costs at the individual, family, and community level, suicide currently outnumbers homicide and motor vehicle accident collisions [1,2], representing a public health emergency and resulting in an estimated cost of $93.5 billion to the U.S. economy [3]. Despite unprecedented strategies to advance awareness and treatment [4,5,6], suicide rates have remained intractable over time and recently increased in some cases, rising by approximately 24% in the U.S. (10.5 to 13/100,000) from 1999–2014 [7]. Alarmingly, the majority of suicide decedents consult with their physician in the days and weeks prior to death [8,9], suggesting missed detection of risk despite intervention opportunity. Moreover, known risk factors currently show relatively poor sensitivity and clinical utility in predicting suicide occurrence [10,11]. Recent research suggests that youth may disclose risk factors for suicide on Facebook or Twitter that they may fail to disclose to physicians, indicating a unique interplay of risk factors that may likewise vary according to age [12]. As a transdiagnostic outcome of medical illness, suicide rates are impacted by a unique interplay of risk factors that may change substantially over time. This includes differences in suicide risk according to age, gender, and ethnicity, which may furthermore vary according to region, suicide method, and access to health care [13,14]. This underscores need for rigorously-designed, population-based investigations of suicide risk, which may face significant cost, clinical, and infrastructural barriers. 

As a form of artificial intelligence (AI), machine learning (ML) methods enable computer learning of advanced classifiers that may improve the accuracy of prediction using large-scale datasets. Given challenges inherent in traditional research methods, including cost and clinical barriers, risk of bias, and restricted generalizability, researchers have begun leveraging large datasets using advanced predictive modeling techniques [15,16,17,18]. This includes the application of ML to electronic medical records (EMR) within modern clinical informatics to advance risk prediction [19]. Risk models learned, using such data, have been developed to predict preterm infant morbidity [20], early warning signs of sepsis [21], and risk for rare outcomes (e.g., first-onset cancers, congestive heart failure, and schizophrenia) with a high degree (i.e., >90%) of accuracy [22]. Such studies indicate that ML approaches can be used to derive appropriate variable weights to produce models that can outperform corresponding, expert-derived scoring systems. On an organizational level, such models have also been used to predict demand for emergency department (ED) beds and elective surgery case volume to inform hospital staffing decisions [23,24,25] and enhance clinical care.

As such, the use of artificial intelligence and machine learning offers new possibilities to significantly guide risk prediction and advance suicide prevention frameworks. Though recent studies yield promising findings [15,26,27,28,29], ML investigations for suicide prevention span diverse medical and computer science fields—challenging ease of review, dissemination, and impact. We therefore conducted a systematic review of empirical reports in this area, with a primary focus on the use of AI in suicide prevention. Our aim was to identify and summarize original reports employing use of an AI/ML framework to predict suicidal behaviors as an outcome of risk according to systematic review.

## 2. Methods 

### 2.1. Search Strategy

A web-based systematic literature search was performed for articles published from inception through November 30, 2018 on PubMed/MEDLINE, EMBASE, PsycINFO, and Web of Science, using search strings pertaining to suicide and ML. Database searches were supplemented by hand-search techniques.

Key words were used by search engine and designated filters according to PRISMA guidelines:

(A). PubMed: (“Artificial Intelligence"[Mesh] OR machine learning[tw] OR natural language processing[tw] OR artificial intelligence[tw]) AND ("Suicide"[Mesh] OR suicid*[tw])
(B). EMBASE: (’artificial intelligence’:ti,ab,kw OR ’machine learning’:ti,ab,kw OR ’natural language processing’:ti,ab,kw) AND (’mood disorder’:ti,ab,kw OR ’depression’:ti,ab,kw OR ’bipolar disorder’:ti,ab,kw OR ’suicidal behavior’:ti,ab,kw OR ’suicide’:ti,ab,kw OR suicid*)
(C). Web of Science: TS = (“artificial intelligence" OR "machine learning" OR "natural language processing") AND TS = ("mood disorder" OR depress* OR bipolar OR suicid*)
(D). PsycINFO: ((“artificial intelligence" or "machine learning" or "natural language processing") and ("mood disorder" or bipolar or depress* or suicid*)).ab,hw,id,ot,ti.

### 2.2. Study Selection

This review was performed according to the EQUATOR/PRISMA guidelines (Enhancing the Quality and Transparency of Health Research/Preferred Reporting Items for Systematic Reviews and Meta-Analyses), which serves as an evidence-based protocol for selecting and reporting for systematic reviews and meta-analyses [30]. Given that suicidal behaviors exist across all ages and diverse medical conditions, diagnostic or age-related variables were not a basis for exclusion.

### 2.3. Data Collection Process

Reviewers (R.A.B., A.M.H.) independently reviewed abstracts, followed by full-text articles. A third reviewer (R.M.) made a final decision, if there was a lack of consensus. Source documents were assessed according to the following inclusion criteria: (1) Journal article (Available in English), (2) original investigation (non-review/commentary), (3) employment of AI/ML methodology, and (4) evaluation of a suicide risk outcome (i.e., defined using CDC-derived guidelines [31] for suicidal self-directed violence; non-suicidal self-injury was excluded), grouped and labeled by suicidal behavior type (e.g., suicide ideation, suicide attempts, suicide death, or other). Studies identified by the above search strategy were managed using Endnote X8. Reports failing to meet inclusion criteria were systematically excluded with reasons. A PRISMA flow chart [30] was created to graphically depict inclusion/exclusion of studies by level of (1) identification, (2) screening, (3) eligibility, and (4) inclusion, and reports were coded with established quality ratings [32]. Reports were further grouped according to suicidal behavior type, sample characteristics, and AI/ML methodology. The latter included use of supervised learning, which aims to predict outcomes based on a set of input values, used to train a classifier; whereas, in unsupervised learning, no labels are provided and the aim is to instead describe data patterns, often by way of clustering, based on input measures. Studies were also coded for use of natural language processing (NLP)—which uses a computer to automatically or semi-automatically process human-generated language—and summarized by other study characteristics, including evaluation of biological markers of suicide risk.

### 2.4. Data Analysis

Descriptive analyses were employed to analyze study findings by key design characteristics according to suicide risk outcome, where ML parameters (e.g., area under the curve (AUC), accuracy, sensitivity, specificity) were reported. The data collated was not amenable to synthesis and meta-analysis was therefore not possible for evaluation.

## 3. Results 

### 3.1. Data Extraction

**Figure 1:**
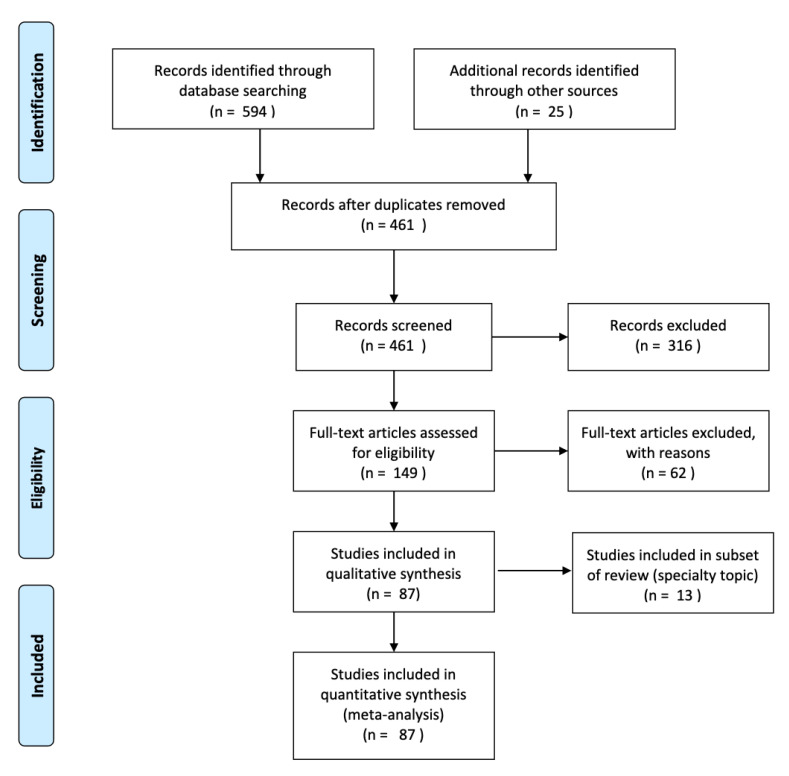
Preferred Reporting Items for Systematic Reviews and Meta-Analyses (PRISMA) flow diagram.

A total of n = 594 records were identified according to the above search methods. An additional 25 records were identified through handsearch and Google Scholar articles. Of n = 461 unique articles, a further n = 316 were excluded according to abstract screening. Full text review was performed for n = 149 articles according to study inclusion criteria. Forty-nine reports failed to meet stated inclusion criteria. This included failure to: represent an original report (i.e., vs. a review/commentary), employ AI/ML methodology, or evaluate a suicide risk outcome (i.e., according to CDC-defined suicidal behaviors). N = 87 studies were included for qualitative synthesis; a subsample of reports (n = 13) met criteria as a subset of this review (see Figure 1). These evaluated emotional content among suicide decedent notes using natural language processing (NLP), and will be discussed separately. A total of n = 87 studies were included in a quantitative analysis. For reports meeting primary review inclusion, this represented an aggregate total number of n = 5,986,238 patients. Sample size was unreported or unavailable in n = 7 studies.

### 3.2. Broad Outcome Groupings

For broad outcome groupings in the quantitative synthesis, a total of n = 42 reports assessed suicide attempt (n = 28) or suicide death (n = 14) as a primary outcome. A total of n = 45 studies evaluated suicidal ideation (i.e., history and current symptoms) (n = 9), multiple risk outcomes (n = 18), and other-social media (n = 10) or other-undifferentiated (n = 8) risk outcomes. 

### 3.3. ML Techniques and Learning Methods

ML methodology varied widely across reports and included both supervised and unsupervised learning algorithms. The majority of studies employed supervised learning techniques, which included ensemble learning methods (e.g., especially random forests), naïve Bayes classification, decision trees, logistic/least squares regression, and support vector machines (SVM). In comparison, n = 7 studies used unsupervised learning techniques, which included clustering algorithms, neural networks, self-organizing maps (SOM), principal component analysis (PCA), and decision trees. Only three studies used both supervised and unsupervised learning methods. Cross-validation techniques, or methods for splitting the data into training and test sets for model performance testing, were variably reported, with few investigations using distinct datasets, separated in time, for training and test models. (See Table 1 for all studies (n = 87). See Table 2 for a subset of reports (n = 13) in this review). 

### 3.4. Design Characteristics

Broadly grouped, several general approaches were visible in study design methodology: (1) Investigations designed to explore the accuracy of diagnostic classification (i.e., using ML techniques and a large dataset or number of variables) to identify those at risk by classifying a binary suicide risk outcome (n = 65) (i.e., classification studies); or (2) investigations evaluating conceptual models of suicide risk, which ranged from PCA and other clustering algorithm methods (e.g., hidden layers, discovering patterns). Prospective designs were used in a small number of studies (n = 21), whereas the majority of investigations used a cross-sectional study design. Several reports (n = 12) utilized a population-based or epidemiologic design, and over half included multi-site investigations. In general, according to the Oxford Centre for Evidence-Based Medicine Protocol [32], articles ranged between ratings of 2–4, with most represented by a 3 rating. A 2 rating describes well-designed, controlled trials without randomization or prospective comparative cohort trials; a 3 rating refers to studies that employ case controls or retrospective cohort investigations; whereas a 4 rating represents case series studies with or without intervention or use of a cross sectional design. No randomized controlled, adequately powered trials (i.e., 1 rating) were identified in this review.

### 3.5. Sample Size and High Dimensional Data

Across all investigations, samples ranged in size from 55 to 975,057 (M = 74,815, SD = 217,839; Md n = 761 participants). While the majority of reports harnessed big data, several studies (n = 6) investigated high dimensional datasets with small sample sizes, which may increase the risk of overfitting. These studies evaluated a large volume of variables (i.e., >400), using smaller samples (i.e., ranging from n = 34–135 participants) to classify and detect differences in risk outcome.

### 3.6. Sample Characteristics

Samples varied significantly in ages studied, with the majority evaluating adults, and a smaller proportion investigating pediatric (n = 26), geriatric (n = 15), or all-age (n = 8) samples. A total of n = 16 studies evaluated risk among military personnel or veterans, and across all reports, the use of a clinical sample was observed in the majority of cases. These included participants recruited from high-risk or triage settings, such as the emergency department (ED) (n = 15). Reports demonstrated a primarily transdiagnostic focus, with few focusing on risk among specific psychiatric conditions, such as mood disorders and schizophrenia (n = 13). Other studies (n = 10) included social media investigations without diagnostic specifiers or assessed an undifferentiated outcome of suicide risk (i.e., suicide risk stratification and clinical decision-making prediction; suicide gene marker detection; human vs. machine learning classification testing, etc.). Finally, the number of studies utilizing electronic medical records (EMR) or administrative chart data was high, particularly in comparison with those using epidemiologic surveys (n = 12) or social media user data (n = 10). Use of a convenience sample or re-evaluation of archival datasets using ML techniques was common in comparison with a priori-designed studies.

### 3.7. Natural Language Processing and Biological Markers of Risk 

Twenty-nine investigations employed the use of natural language processing (NLP) in association with suicidal behaviors. These included investigations evaluating (n = 2) acoustic features of speech to identify risk within emergency department settings, text-based applications (n = 1), or investigation of social media user data or posts (n = 15). A few such studies generated a word map to note word frequency in association with risk within EMR, medical discharge notes, or social media posts. A small number of ML investigations (n = 12) evaluated a biological marker of risk, such as plasma and blood metabolites (n = 2), genes (n = 8), and neuroimaging (n = 1), to predict risk for suicidal behaviors and hospitalization.

### 3.8. Timeframe of Assessment and Predictive Modeling

Timeframe of risk detection was variable, ranging from the next 24 h to lifetime assessments of suicide outcomes. Where reported, the majority (n = 15) investigated suicide risk prediction over a monthly timeframe. This ranged from 1 to 24 months (n = 9.31). Two reports investigated risk over an acute timeframe (24–72 h), and n = 12 studies evaluated lifetime risk. Four investigations reported multiple timeframes of risk, whereas n = 21 failed to report or specify precise observation or time-at-risk periods.

### 3.9. Accuracy, Area Under the Curve, Positive Predictive Value, Sensitivity/Specificity

**Figure 2:**
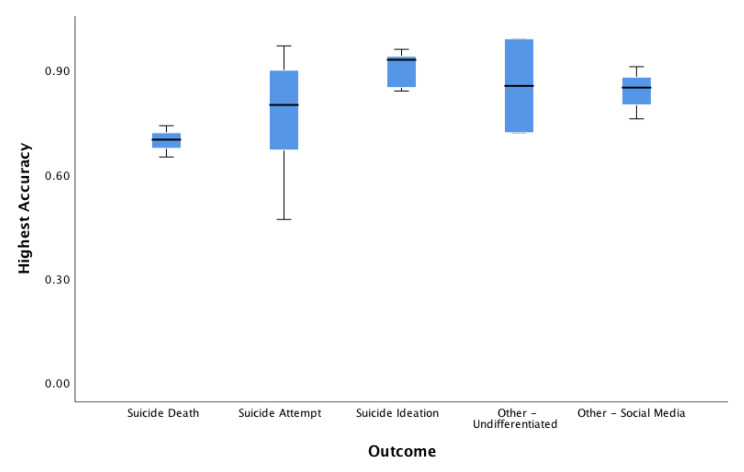
Boxplot of accuracy by suicide outcome. Boxplot of classification accuracy by suicide outcome groupings. Notes: Outcomes assessed suicide death (M = 0.69 (SD = 0.04)); 95% CI (0.58, 0.80), suicide attempt (M = 0.82 (SD = 0.12)); 95% CI (0.71, 0.92), suicide ideation (M = 92 (SD = 0.04)); 95% CI (0.84, 0.99), other-undifferentiated (M = 0.85 (SD = 0.19)); 95% CI (−0.86, 2.57), other-social media (M = 0.84 (SD = 0.04)); 95% CI (0.74, 0.94). CI = confidence interval by outcome.

**Figure 3:**
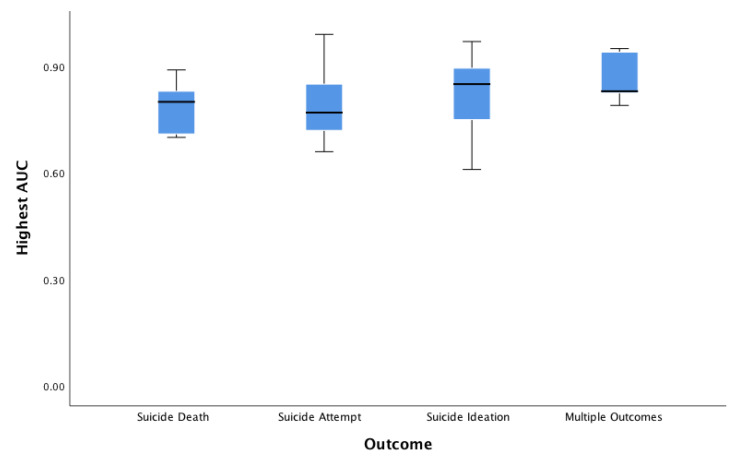
Boxplot of AUC by suicide outcome. Boxplot of classification area under the curve (AUC) by suicide outcome groupings. Notes: Outcomes assessed suicide death (M = 0.79 (SD = 0.13)); 95% CI (−0.41, 2.0), suicide attempt (M = 0.81 (SD = 0.09)); 95% CI (0.76, 0.87), suicide ideation (M = 0.78 (SD = 0.15)); 95% CI (0.52, 1.03), multiple outcomes (M = 0.87 (SD = 0.08)); 95% CI (0.67-1.06). CI = confidence interval by outcome.

Of N = 65 classification studies, a total of n = 41 investigations reported area under the curve (AUC) (or provided sufficient information for this value to be derived; n = 4 cases), (M = 0.814, SD = 0.110, Mdn = 0.820, range 0.61–0.99). In comparison, n = 29 investigations reported accuracy, (M = 0.813, SD = 0.123, Mdn = 0.840, range = 0.47–0.99). Other metrics, such as positive predictive value (PPV), were infrequently reported. PPV was reported in n = 6 cases, (SD = 0.30, Mdn = 0.88, range = 0.18–1.00). In total, n = 31 studies reported sensitivity, (M = 0.749, SD = 0.195, Mdn = 0.790, range = 0.22–1.00), and n = 32 reported specificity, (M = 0.870, SD = 0.156, Mdn = 0.870, range = 0.57–1.00). According to an exploratory one-way analysis of variance (ANOVA) to evaluate non-weighted accuracy and AUC values across reports, significant mean differences were not detected according to type of suicide-related outcome for highest accuracy (F _4,15_ = 1.98, p = 0.149) or AUC (F _5,19_ = 1.52, p = 0.231). See Figure 2 and Figure 3.

## 4. Discussion 

Eighty-seven reports were identified in this systematic review, which included a subset of investigations evaluating emotional sentiment among suicide notes using ML methods. Across reports meeting primary inclusion criteria, the majority of studies examined risk for suicide attempts, followed by death by suicide, suicidal ideation, and multiple risk outcomes. A small proportion of studies predicted risk of an outcome in-between these groupings, including those examining an undifferentiated outcome (e.g., unspecified suicidal behavior, or ”suicidality”) or harnessing social media data (e.g., suicide-related risk by Twitter or internet post content) [85,86,87,88,89,90,91,92]. Based on this review, use of AI/ML methods for suicide risk prediction is a burgeoning area of inquiry, reflected by the diversity of fields represented and the pace of publications. Though 1999 marked the earliest publication, nearly half of reports were published in the past three years. This suggests an area of rapid growth at a nascent stage of investigation, presenting opportunities to critically guide the field forward and address key gaps in the extant literature.

Machine learning methods varied substantially across studies and ranged significantly in rigor and model testing. Supervised learning was most commonly used compared to unsupervised learning techniques, and few studies used both methods. In general, exploratory investigations were overrepresented, and replication or application of a predictive model—within a new setting or sample—was rare. Several reports tested replication in a new cohort—within the same setting—or used a multiple-wave sampling approach [15,26,34,97]. Methodologically, these represent critical areas of importance for future studies and warrant replication. Classification studies were most commonly observed in this review, and excellent accuracy and area under the curve (AUC) values were observed, despite considerable differences in design, methodology, sample, and learning methods. Model performance metrics most frequently reported were AUC, whereas accuracy, sensitivity, and specificity were reported in less than a third of reports. According to broad outcome groupings, underreporting and low cell counts by outcome groupings challenge interpretation and adequately powered comparisons.

Regarding generalizability, reports reflected a transdiagnostic focus, and primarily assessed adult participants or patient records. A smaller number of reports examined high-risk, pediatric or geriatric samples, as well as military veterans [15,26,33,34,53,72,97,112]. These highlight areas of elevated need, and align with prioritized strategies and nationally-directed initiatives for technology innovation in suicide prevention [5,6,130]. Investigations predominantly evaluated clinical samples or emergency settings, consistent with increased risk post-hospitalization [15,26,34,43,68,131]. Regarding constraints, archival datasets were common, with fewer studies employing prospective data elements [15,34,55,106]. Though convenience samples present inherent limitations, this has likewise been emphasized as a relative strength—insofar as ML may be applied to large-scale datasets that, as yet, remain unstudied [34]. This highlights opportunity for re-analysis of existing datasets to advance early detection and prevention methods, where prospective samples warrant prioritization. Next, though several reports used epidemiologic surveys within nationally-representative sample [60,101,106]. Such surveys, however, frequently used a single item assessment of suicidal behaviors, which may misclassify risk [132,133]. In general, suicide outcomes were variably defined, and validated symptom instruments varied significantly across reports [26,56,57,59,72,78,86,95,102,103]. This aligns with calls for increased uniformity in the assessment of suicidal behaviors to enhance research comparisons and improve surveillance [31]. We recommend that such calls be applied to the study of suicidal behaviors across ML investigations to enhance uniformity, comparison, and opportunity to improve risk prediction frameworks.

In several cases, the development and testing of clinical prediction models were evaluated against traditional statistics [6,55], showing superiority of ML in the classification of risk. Reports likewise compared ML-guided decision tree models to clinician-based predictions (i.e., of hospitalization following a suicide attempt (SA) or predicting likelihood of a suicide risk outcome) to guide triage [6,55,59,76,78,96]. Importantly, ML-guided risk stratification models outperformed those relying on clinician-based prediction methods alone. This included model testing within acute time frames of risk (i.e., 3–6 months) [55,96]—in one case, with performance enhanced three-fold using ML risk stratification [96]. Such findings suggest that advanced data analytic methods, combined with computer-guided screening, may augment clinical decision-making. Replication is warranted, including how such models may guide triage to optimize patient care with minimal time burden to providers. 

Given that the majority of suicide decedents consult with their physician prior to death [8,9], such methods hold promise to enhance early detection and opportunity for rapid intervention. This may be particularly relevant to emergency settings, where medical records have been compared with manual coding of suicide attempt encounters using machine learning with promising results [43]. This aligns with research suggesting that brief, low-risk suicide prevention strategies targeting emergency settings are both efficacious and cost-effective [134,135,136]. The way suicidal behaviors are coded within EMR may likewise pose challenges to risk detection. Anderson and colleagues [102] used ML to evaluate correspondence between patient notes and ICD/E-Codes (International Classification of Disease/ICD External Cause of Injury Code) for suicidal behaviors, based on text-mining of clinical discharge notes in a sample of n = 15,761 patient records. They observed a low level of correspondence, with only 3% of encounters coded for suicidal ideation and 19% coded for suicide attempts. This suggests nned for considerable caution when interpreting suicide risk using ICD/E-Codes from EMR data alone, in comparison with discharge notes. 

A subset of studies investigated NLP as a novel area of inquiry in select settings or populations. Pestian et al. [26] investigated NLP (i.e., key words and vocal characteristics) in structured and free-text speech responses to accurately distinguish (96.6% accuracy) n = 60 youth presenting to an ED for suicide risk (i.e., versus those presenting for other reasons). Text-mining methods also predicted accurate classification of those at risk for later suicidal behaviors [109,110,112,113], in some cases, generating word maps that may aid future research. Other novel approaches included social media investigations of microblog users and Twitter posts to detect suicide risk among users, online communities, or posts following a natural disaster to index public emotion [66,85,86,87,88,89,105]. Despite a large number of neuroimaging and neuroanatomical reports within suicide prevention, a smaller number of studies examined a biological variable in this review. Baca-Garcia and colleagues [56] showed that an algorithm based on three CNS (Central Nervous System) single nucleotide polymorphisms (SNPs) correctly classified those with and without a suicide attempt history, whereas other investigations evaluated candidate biomarkers to predict future risk for suicide [64,69,103]. Only one study used neuroimaging—comparing youth with suicidal ideation (n = 17) to matched controls (n = 17) on fMRI variables [74]. Based on neural representations in response to suicide and death-related scan stimuli, this generated a high (91%) classification accuracy [74]. This signals a promising approach to biomarker discovery, underscoring integration of biological, behavioral, and clinical variables to inform etiology and intervention in an area with few selective treatments [137,138].

### Critical Challenges and Future Directions

A number of limitations should be noted. Methods varied widely across reports, both with respect to ML methods and study quality. Despite considerable diagnostic and methodological heterogeneity, high levels of model performance were observed. Incomplete reporting of test statistics (e.g., accuracy, AUC, sensitivity, specificity) and differing methods for assessing and defining risk within diverse ML methods—highlights need for improved reporting standards and a priori-designed studies. Key parameters, such as PPV, area under the precision curve (AUPRC), and lead-time of the prediction—which allows for decision-making about when to potentially act and intervene—were also underreported. Challenges inherent in retrospectively analyzing health data for administrative and clinical purposes should also be noted, given the high number of studies using EMR. Hersh et al. [139] raised concerns regarding biases due to EMR data being collected only at hospital visits, incomplete records or missing data, and other considerations relevant to accurate coding that emphasize advanced statistical methods be used for correction. Others report similar concerns of omission in EMR, calling for longitudinal measurements [19]. Additionally, given the way in which differences in the splitting of training data may alter the performance of predictive modeling [140], use of multiple methods to separate samples (i.e., for training versus testing of algorithms) is recommended. Critically, the majority of studies were cross-sectional in nature, underscoring need for prospectively-designed ML investigations to advance suicide risk prediction.

A lack of application to new settings or populations also highlights need for replication, particularly according to longitudinal, well-defined outcomes of risk. Though translation of one model to a new site poses inherent challenges, a model can be trained with data from any local site and tested using data from the site itself [23]. Regarding future application, challenges in constructing and deploying a statistical model within a clinical setting include access to data, availability of skilled personnel, and need to identify ways of integrating the model into healthcare workflows [23]. Others have emphasized associations between model complexity and predictive accuracy [141], in addition to key limitations [142]. For example, Siddaway and colleagues [142] suggest that ML may be best harnessed when led by clinical need, becoming machine-assisted learning similar to other statistical techniques, cautioning against over-reliance on ML models. We recommend incorporation of these considerations into the design of new investigations utilizing machine learning in the detection and prediction of risk for suicidal behaviors.

## 5. Conclusions 

In conclusion, findings of this review highlight risk factors that align with past non-ML findings (e.g., mood/substance disorders, male gender, family history, previous hospitalization, unemployment, comorbidity, and delinquency); whereas, newly-identified risk variables or approaches point to sleep, circadian, and neural substrates, and NLP-derived indices of speech or user data. These findings reflect a burgeoning literature that warrant future study in an area of prevention prioritized worldwide. Though a leading cause of death, suicide defies prediction given its rare occurrence at the population level, which poses important challenges to prevention. AI and ML applications hold unique promise to enable precision medicine in the prevention of suicide, particularly given their ability to handle large and complex datasets. We propose that such methods may crucially inform the early detection of suicide risk, triage, and treatment development, with important methodological and statistical cautions. The application of NLP to social media in particular, and integration of AI with real-time suicide risk assessments, holds unique promise to impact the prevention of suicide on a broad scale.

## Figures and Tables

**Table 1:** Investigations by broad outcome groupings and ML parameters.

Author	Year	Journal	Quality Rating		Clinical Sample	Outcome	Biomarker	NLP ^a^		Classification	Specificity	Sensitivity	Accuracy	AUC ^b^	N
Kessler et al. [33]	2017	Int. J. Methods Psychiatr. Res.	2		x	1				x		0.28			6360
McCoy et al. [27]	2016	JAMA Psychiatry	3		x	1			x	x					458,053
Kessler et al. [34]	2017	Mol. Psychiatry	2		x	1				x	0.95	0.46	0.70	0.70	975,057
Kessler et al. [15]	2015	JAMA Psychiatry	3		x	1								0.89	53,769
Poulin et al. [35]	2014	PLoS One	4		x	1	x		x	x			0.65		210
Pestian et al. [36]	2008	AMIA Annu. Symp. Proc.	3			1			x	x			0.74		66
Pamer et al. [37]	2008	AMIA Annu. Symp. Proc.	3			1				x					1204
Haerian et al. [38]	2012	AMIA Annu. Symp. Proc.	3		x	1			x	x					280
Ilgen et al. [39]	2009	J Clin. Psychiatry	3		x	1									887,859
Adamou et al. [40]	2019	Crisis	3		x	1			x	x				0.70	130
Rossellini [41]	2018	Depress. Anxiety	3		x	1				x				0.86	9488
De Avila Berni [42]	2018	PLOS One	5			1				x	0.91	0.69		0.80	
Metzger et al. [43]	2017	Int. J. Methods Psychiatr. Res.	3			2			x	x					444
Passos et al. [44]	2016	J. Affect. Disord.	4		x	2					0.71		0.72	0.77	144
Kessler et al. [45]	2016	Mol. Psychiatry	2			2				x		0.70		0.76	1056
Modai et al. [46]	2004	J. Nerv. Ment. Dis.	2		x	2					0.85	0.94			987
Modai et al. [47]	2002	JMIR Med. Inform.	3		x	2					0.85	1.00		0.82	197
Modai et al. [48]	1999	Med. Inform. Internet Med.	3			2					0.94	0.94		0.94	198
Modai et al. [49]	2002	Crisis	2		x	2									250
Modai et al. [50]	1998	Med. Inform.	4		x	2	x				0.97	0.83			161
Hettige et al. [51]	2017	Gen. Hosp. Psychiatry	3		x	2	x			x	0.8	0.65	0.67	0.71	345
Walsh et al. [52]	2017	Clin. Psychol. Sci.	3		x	2				x		0.96		0.84	5167
Venek et al. [53]	2017	IEEE Trans. Affect. Comput.	3		x	2				x			0.90		60
Baca-Garcia et al. [54]	2007	Prog. Neuropsych.. Biol Psychiatry	4		x	2	x			x	0.99	0.99	0.97	0.99	539
Tiet et al. [55]	2006	Alcohol Clin. Exp. Res.	3		x	2	x		x	x	0.87	0.89		0.88	34,251
Baca-Garcia et al. [56]	2010	Am. J. Med. Genet. B Genet.	3		x	2	x			x	0.82	0.50	0.67	0.66	277
Lopez-Castroman et al. [57]	2011	J. Psychiatry Res.	4		x	2	x			x	0.97		0.76	0.71	1349
Modai et al. [58]	2004	Med. Inform. Internet Med.	3		x	2					0.70	0.83		0.77	612
Mann et al. [59]	2008	J. Clin. Psychiatry	3		x	2				x	0.92	0.89		0.80	408
Bae et al. [60]	2015	Neuropsychiatr. Dis. Treat.	3			2				x					2754
Choo et al. [61]	2014	Asian J. Psychiatr.	3		x	2							0.90		418
Oh et al. [62]	2017	Front. Psychol.	3		x	2				x	0.99	0.78	0.97		573
Benton et al. [63]	2017	Proc. 15th Conf. EACL	3		x	2				x					9611
Ruderfer et al. [64]	2019	Mol. Psychiatry	3			2	x				0.82	0.92		0.94	512,639
Lyu and Zhang [65]	2019	J. Affect. Disord.	3		x	2					0.94	0.68		0.85	1318
Coppersmith et al. [66]	2018	Biomed. Inform. Insights	3			2,6			x	x				0.94	418
Dargel et al. [67]	2018	Acta Psychiatr. Scand.	3		x	2	x						0.84		635
Jordan et al. [68]	2018	Psychiatry Res.	3		x	2				x	0.69	0.79		0.72	218
Setoyama et al. [69]	2016	PLoS One	2		x	3	x			x				0.70	90
Pestian et al. [70]	2017	Suicide Life Threat. Behav.	2		x	3			x	x			0.93	0.85	379
Cook et al. [71]	2016	Comput. Math Meth. Med.	2		x	3			x	x	0.57	0.56	0.85	0.61	1453
Pestian et al. [26]	2016	Suicide Life Threat. Behav.	2		x	3			x	x			0.96	0.97	60
Gradus et al. [72]	2017	J. Trauma Stress	4		x	3				x				0.92	2240
Birjali et al. [73]	2017	Procedia Comput. Sci.	3			3			x	x					
Just et al. [74]	2017	Nat. Hum. Behav.	3		x	3	x			x			0.94		79
Ryu et al. [75]	2018	Psychiatric Invest.	3			3				x	0.81	0.84		0.80	11,628
Desjardins et al. [76]	2016	J. Clin. Psychiatry	4		x	4				x				0.93	879
Tzeng [77]	2006	Worldview Evid. Base. Nurs.	2		x	4				x					63
Baca-García et al. [78]	2006	J. Clin. Psychiatry	3		x	4				x	1.00		0.99		509
Quan et al. [79]	2014	PLoS One	2			4	x								
Litvinova et al. [80]	2017	Comput. y Sistemas	3		x	4			x	x			0.72		1000
Zhang et al. [81]	2019	Health Inform. J.	3		x	4			x	x					409
McKernan et al. [82]	2019	Arthritis Care Res.	3		x	2,3				x	1.00	1.00		0.82	8879
DelPozo-Banos et al. [83]	2018	JMIR Ment. Health	3		x	1				x	0.85	0.65		0.80	2604
Burke et al. [84]	2018	Psychiatry Res.	3			4				x				0.89	359
Cheng et al. [85]	2017	J. Med. Internet Res.	4			5			x	x				0.61	974
Braithwaite et al. [86]	2016	JMIR Ment. Health	4			5				x	0.97	0.53	0.91		135
Guan et al. [87]	2015	JMIR Ment. Health	4			5				x					909
Woo et al. [88]	2015	Int. J. Environ. Res. Public Health	4			5			x						
O’Dea et al. [89]	2015	Internet Interv.	3			5				x			0.76		14,701
Nguyen et al. [90]	2017	Multimed. Tools Appl.	3	x		5				x			0.88		
Burnap et al. [91]	2017	Online Soc. Netw. Media	3			5			x	x			0.85		
Vioules et al. [92]	2018	IBM J. Res. Dev.	3			5			x	x					120
Zalar et al. [93]	2018	Psychiatr. Danub.	3			1, 2				x			0.91		78,625
Tran et al. [94]	2015	J. Biomed. Inform.	3	x		1,2				x					7578
Leiva-Murillo et al. [95]	2013	Comput. Math. Methods Med.	3	x		1,2	x								8699
Tran et al. [96]	2014	BMC Psychiatry	3	x		1,2					0.97			0.79	7399
Bernecker et al. [97]	2019	Behav. Res. Ther.	3	x		2, 3				x				0.83	27,501
Zhong et al. [98]	2019	Euro. J. Epidemiol.	3			2, 3			x	x	0.96	0.34		0.83	275,843
Morales et al. [99]	2017	Front. Psychiatry	4	x		2,3				x	0.79		0.71		707
Barros et al. [100]	2017	Braz. J. Psychiatry	4	x		2,3				x	0.79	0.77	0.78		707
Kuroki [101]	2015	Am. J. Orthopsychiatry	3			2,3			x	x					624
Anderson et al. [102]	2015	J. Am. Board Fam. Med.	3	x		2,3,4			x	x	0.96	0.94		0.95	15,761
Levey et al. [103]	2016	Mol. Psychiatry	3	x		1,3,4	x							0.94	114
Colic et al. [104]	2018	Conf. Proc. IEEE Eng. Med. Biol. Soc.	3	x		3							0.84		738
Aladag et al. [105]	2018	J. Med. Internet Res.	3			5			x	x			0.92		785
Choi et al. [106]	2018	S. Korean J. Affect. Disord.	3			1				x				0.72	819,951
Downs et al. [107]	2018	AMIA Annu. Symp. Proc.	3	x		4			x	x					1906
Fahey [108]	2018	Soc. Sci. Med.	3			5			x	x			0.80		974,891
Zhong et al. [109]	2018	BMC Med. Inform. Decis. Mak.	3	x		2-4			x	x					275,843
Fernandes et al. [110]	2018	Sci. Rep.	3			2,3			x	x		0.87			
Jordan et al. [111]	2018	Gen. Hosp. Psychiatry	3	x		3			x		0.83			0.87	6805
Carson et al. [112]	2019	PLOS one	3	x		2			x	x	0.22	0.83	0.47	0.68	73
McCoy et al. [113]	2018	Depress. Anxiety	3			1			x						444,317
Connolly et al. [114]	2017	BMC Bioinform.	3	x		3				x					314
Modai et al. [115]	2002	Euro. Psychiatry	4	x		2					0.73	0.65			250
Rossellini et al. [116]	2017	Psychol. Med.	3			2				x				0.74	21,832

Investigations by broad outcome groupings and ML parameters. Outcomes: 1 = suicide death, 2 = suicide attempt/medically serious suicide attempt, 3 = suicidal ideation/state, 4 = other-undifferentiated, 5 = other-social media risk outcomes. Notes: NLP = natural language processing a; AUC = area under the curve b; empty cells indicate missing/unreported data in articles; ML = machine learning.

**Table 2:** Investigations by study subset and ML parameters.

Citation	Journal	Outcome	Precision	Quality Rating
Liakata et al. 2012 [117]	Biomed Inform Insights	Death/NLP of Suicide Notes	0.60	3
Nikfarjam et al. 2012 [118]	Biomed Inform Insights	Death/NLP of Suicide Notes	0.60	3
Yeh et al. 2012 [119]	Biomed Inform Insights	Death/NLP of Suicide Notes	0.77	3
Cherry et al. 2012 [120]	Biomed Inform Insights	Death/NLP of Suicide Notes	1.00	3
Wang et al. 2012 [121]	Biomed Inform Insights	Death/NLP of Suicide Notes	0.67	3
Desmet et al. 2012 [122]	Biomed Inform Insights	Death/NLP of Suicide Notes	NR	3
Kovacevic et al. 2012 [123]	Biomed Inform Insights	Death/NLP of Suicide Notes	0.67	3
Pak et al. 2012 [124]	Biomed Inform Insights	Death/NLP of Suicide Notes	0.62	3
Spasic, 2012 [125]	Biomed Inform Insights	Death/NLP of Suicide Notes	0.55	3
McCarthy et al. 2012 [126]	Biomed Inform Insights	Death/NLP of Suicide Notes	0.57	3
Wicentowski et al. 2012 [127]	Biomed Inform Insights	Death/NLP of Suicide Notes	0.69	3
Sohn, 2012 [128]	Biomed Inform Insights	Death/NLP of Suicide Notes	0.61	3
Yang, 2012 [129]	Biomed Inform Insights	Death/NLP of Suicide Notes	0.58	3

Investigations (N = 13) by study subset and ML parameters. Outcome focused on sentiment detection of suicide decedent notes using NLP. Notes: Quality ratings were performed according to the Oxford Centre for Evidence-Based Medicine Protocol; ML = machine learning; NLP = natural language processing; Precision = positive predictive value (PPV); NR = not reported.
